# *In Silico* Pelvis and Sacroiliac Joint Motion: Refining a Model of the Human Osteoligamentous Pelvis for Assessing Physiological Load Deformation Using an Inverted Validation Approach

**DOI:** 10.1155/2019/3973170

**Published:** 2019-01-09

**Authors:** Maziar Ramezani, Stefan Klima, Paul Le Clerc de la Herverie, Jean Campo, Jean-Baptiste Le Joncour, Corentin Rouquette, Mario Scholze, Niels Hammer

**Affiliations:** ^1^Auckland University of Technology, Department of Mechanical Engineering, Auckland, New Zealand; ^2^Department of Anatomy, University of Otago, Dunedin, New Zealand Department of Anatomy, Dunedin, New Zealand; ^3^Department of Trauma, Orthopedic and Plastic Surgery, University Hospital of Leipzig, Germany; ^4^Supméca-Institut Supérieur de Mécanique de Paris, Paris, France; ^5^Fraunhofer Institute for Machine Tools and Forming Technology, Dresden, Germany

## Abstract

*Introduction. *Computational modeling of the human pelvis using the finite elements (FE) method has become increasingly important to understand the mechanisms of load distribution under both healthy and pathologically altered conditions and to develop and assess novel treatment strategies. The number of accurate and validated FE models is however small, and given models fail resembling the physiologic joint motion in particular of the sacroiliac joint. This study is aimed at using an inverted validation approach, using* in vitro* load deformation data to refine an existing FE model under the same mode of load application and to parametrically assess the influence of altered morphology and mechanical data on the kinematics of the model.* Materials and Methods. *An osteoligamentous FE model of the pelvis including the fifth lumbar vertebra was used, with highly accurate representations of ligament orientations. Material properties were altered parametrically for bone, cartilage, and ligaments, followed by changes in bone geometry (solid versus 3 and 2 mm shell) and material models (linear elastic, viscoelastic, and hyperelastic isotropic), and the effects of varying ligament fiber orientations were assessed.* Results. *Elastic modulus changes were more decisive in both linear elastic and viscoelastic bone, cartilage, and ligaments models, especially if shell geometries were used for the pelvic bones. Viscoelastic material properties gave more realistic results. Surprisingly little change was observed as a consequence of altering SIJ ligament orientations. Validation with* in vitro* experiments using cadavers showed close correlations for movements especially for 3 mm shell viscoelastic model.* Discussion. *This study has used an inverted validation approach to refine an existing FE model, to give realistic and accurate load deformation data of the osteoligamentous pelvis and showed which variation in the outcomes of the models are attributed to altered material properties and models. The given approach furthermore shows the value of accurate validation and of using the validation data to fine tune FE models.

## 1. Introduction

The finite element (FE) method has become a valuable tool to examine the human pelvis under a variety of conditions, which may include physiological load distribution and the mechanisms of injury [1–10], as well as treatment of pelvic pain and injury [11–16]. Furthermore, FE simulations can be rerun in a highly reproducible manner and therefore can be used to run so-called parametric analyses, tests to examine the effects of changes in material properties, geometries, or boundary conditions. In contrast, cadaver experiments have the disadvantages of limited tissue availability and interindividual variation to varying extent.

In order to provide meaningful results, precise anatomy, realistic load deformation data of pelvic bones, cartilage, and ligaments as well as realistic loading conditions the FE model is exerted to, are crucial. While morphological data for the osteoligamentous pelvis have become readily available [17–22], there is an evident lack of accurate mechanical data, in particular of the soft tissues of the pelvis [23, 24]. As a consequence, material data are commonly estimated for FE models. Given that the ligaments, muscles, and fascial structures have multidirectional fiber orientations [17–20, 25–27], it remains even more challenging to model them in an appropriate manner. As a consequence, the results of FE analyses may vary vastly both between* in silico* and comparing* in silico* with* in vitro* data [2, 11, 28].

Previous FE models, including the ones from our group [1, 2, 11], have evolved to include an increasing level of morphological detail. Validation with cadaveric experiments, however, remains an uncommon approach to validate or to adjust the results from numerical simulation. In recent cadaveric experiments combining physiological loading via the fifth lumbar vertebra and both hip joints we have obtained data (*in press*) in a setup identically resembling a given FE model [11], which may now form the basis for validation and parametric adjustment using an inverted validation approach. This coexisting physiological loading scenario in both the cadaver tests and FE modeling opens the opportunity to refine the existing FE models, changing material properties, material laws for bones, cartilage, and ligaments parametrically and to assess the influence of ligament fiber orientations on the deformation behavior of the human osteoligamentous pelvis.

We hypothesized that** (A)** material laws outweigh the effects of mechanical properties and chosen geometry for bone (shell versus solid model) and that** (B)** assigning viscoelastic material properties gives more realistic deformation data compared to linear elastic. The primary outcome was to increase the accuracy of the existing FE model.

It could be shown that altered material properties for bones, cartilage, and ligaments only had little absolute influence on pelvic motion. Deformation was larger using viscoelastic material properties compared to linear elastic and modeling of the pelvis as a 2 mm or 3 mm bone shell model gave much closer results compared to solid bone models. Interestingly, alterations in fiber orientation of the interosseous and posterior sacroiliac ligaments caused little change in the deformation behavior of the pelvis.

## 2. Materials and Methods

### 2.1. Model Geometry, Mesh Generation, and Boundary Conditions

A previously generated FE model of the human osteoligamentous pelvis was refined for the given experiments [11]. The model was based on a 29-year-old male, 185 cm tall, 69 kg weighing who underwent computed tomography (CT) imaging with no pathology as a diagnosis. Bones segmentation was conducted semiautomatically from the CT data using AMRIA 3.1.1 (VSG, Burlington, MA, USA), including the fifth lumbar vertebra, both innominate bones, the sacrum, and the proximal ends of both femora. The bone and cartilage geometries were transferred into solid parts using Geomagic Studio (Morrisville, NC, USA). Conversion into 2 and 3 mm shell models was accomplished using SolidWorks (Dassault Systèmes SolidWorks Corp., Vélizy-Villacoublay, France). Cartilage was modeled for the L5-S1 intervertebral disk, the bilateral auricular sacroiliac joint (SIJ) cartilage, the pubic symphysis, and both hip joints. All resulting geometries were then transferred into Ansys (version 16.2, ANSYS, Inc., Canonsburg, PA, USA). A total of 210 spring elements were included for the main fiber directions of the iliolumbar, sacroiliac, sacrospinous, and tuberous ligaments as well as the obturator membrane and anterior and posterior longitudinal ligaments. While the number of spring elements was kept the same as in a previous study [11], fiber orientations of the sacroiliac joint ligaments were thoroughly revised in particular for the interosseous and posterior SIJ ligaments and refined in consistency with further anatomical cadaveric dissection studies. Meshing was conducted as shown previously using tetrahedral elements [11], with a model containing the number of nodes and elements given in Table 1. Boundary conditions were chosen as follows: 300 N load applied via L5 in the craniocaudal direction and 100 N load applied via each of the femora into both acetabula.

### 2.2. Parametric Analyses and Change in the Material Models

Three major variables were assessed for bone, cartilage, and ligaments:

- *Material properties* including elastic modulus and Poisson ratio: properties were chosen as given in Table 2. The values were varied parametrically, mostly in a range of 50% to 200% of the assumed mean value of the material property
- *Model geometry* for bones: solid, 2 mm shell, and 3 mm shell [37]
- *Material models*: linear elastic, viscoelastic, and hyperelastic (cartilage only)
- *Ligament orientation* (interosseous and posterior sacroiliac joint ligaments)

A linear viscoelastic material model was used for bone and cartilage and the Prony series coefficients were determined from rate-dependent data reported in literature [38, 39]. Further details on the changes in material properties are given in the results section. Pelvis deformation was defined as the motion occurring between L5 and the acetabulum and SIJ deformation as the average motion between the midanterior aspects of the bilateral SIJ.

### 2.3. Measurement Points and Experimental Validation

**Figure 1:**
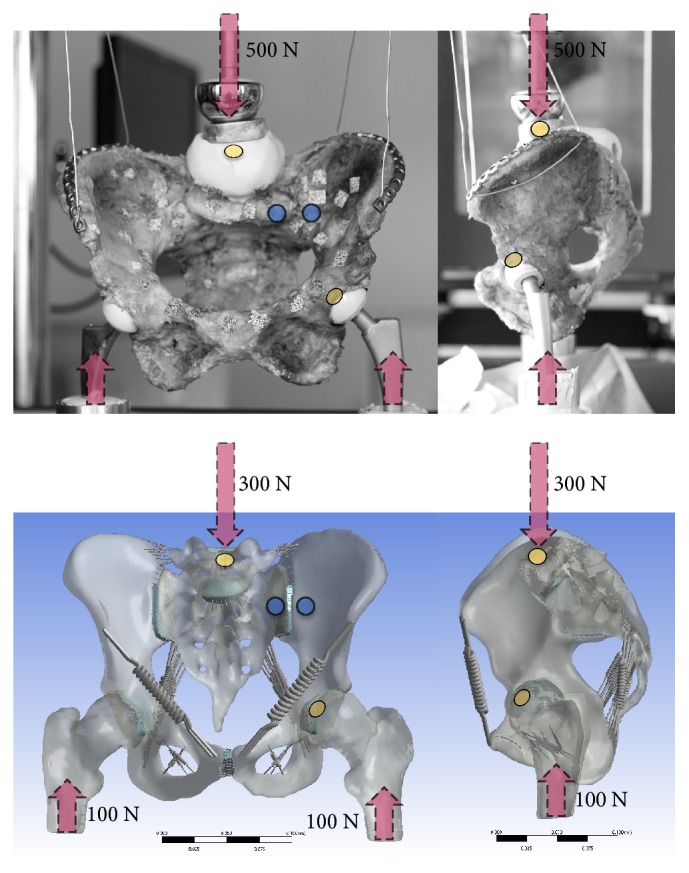
Anterior (top left) and left lateral (top right) view of the loading conditions in the cadaveric pelvises in the cadaveric experiments and in the in silico modeling (bottom). The yellow points indicate the areas where pelvis deformation was assessed, the blue points indicate the areas where SIJ deformation was measured (both exemplarily for the left side).

For parametric analyses, deformations were investigated for the entire pelvis and for the sacroiliac joint under 500 N load application. The results of the numerical analyses were furthermore compared to cadaver experiments (mean age 81.3 ± 10.0 years, range 65-96 years) using the same axial load application, as shown in Figure 1. Here, a two-leg stance setup was simulated, applying loading via the L5 vertebral body, allowing for a physiologic load distribution via the lumbosacral transition and the adjacent ligaments. A uniaxial material testing machine (DYNA-MESS, Aachen, Germany) was applied, and loading was conducted via a spherical stamp component connected to the material testing machine. Femoral head components adapted for each individual acetabulum were used for the mounting to the bottom plates of the testing machine (Figure 1). Following 20 preconditioning cycles at 100 N/sec, load deformation tests were carried out with 100 percent of the individual cadavers' body weight. The load deformation data were additionally obtained using synchronous digital optical image correlation (Limess, Krefeld, Germany) with 2.0 megapixels at 5 fps. For further comparison, the 500-N load step was used from the male pelvises, in line with the* in silico* experiments shown here. Further information on this novel physiologic loading scenario can be found in Hammer et al. and Klima et al. [40, 41]. Data were retrieved for the deformation of the entire pelvis and the SIJ bilaterally, as outlined by the markerpoints shown in Figure 1.

### 2.4. Descriptive Evaluation

The resulting data was plotted using Microsoft Excel version 16.13 (Redmond, WA, USA).

## 3. Results

### 3.1. Bone

#### 3.1.1. Isotropic Linear Elastic Material Properties: Parametric Change of Bone Properties

**Figure 2:**
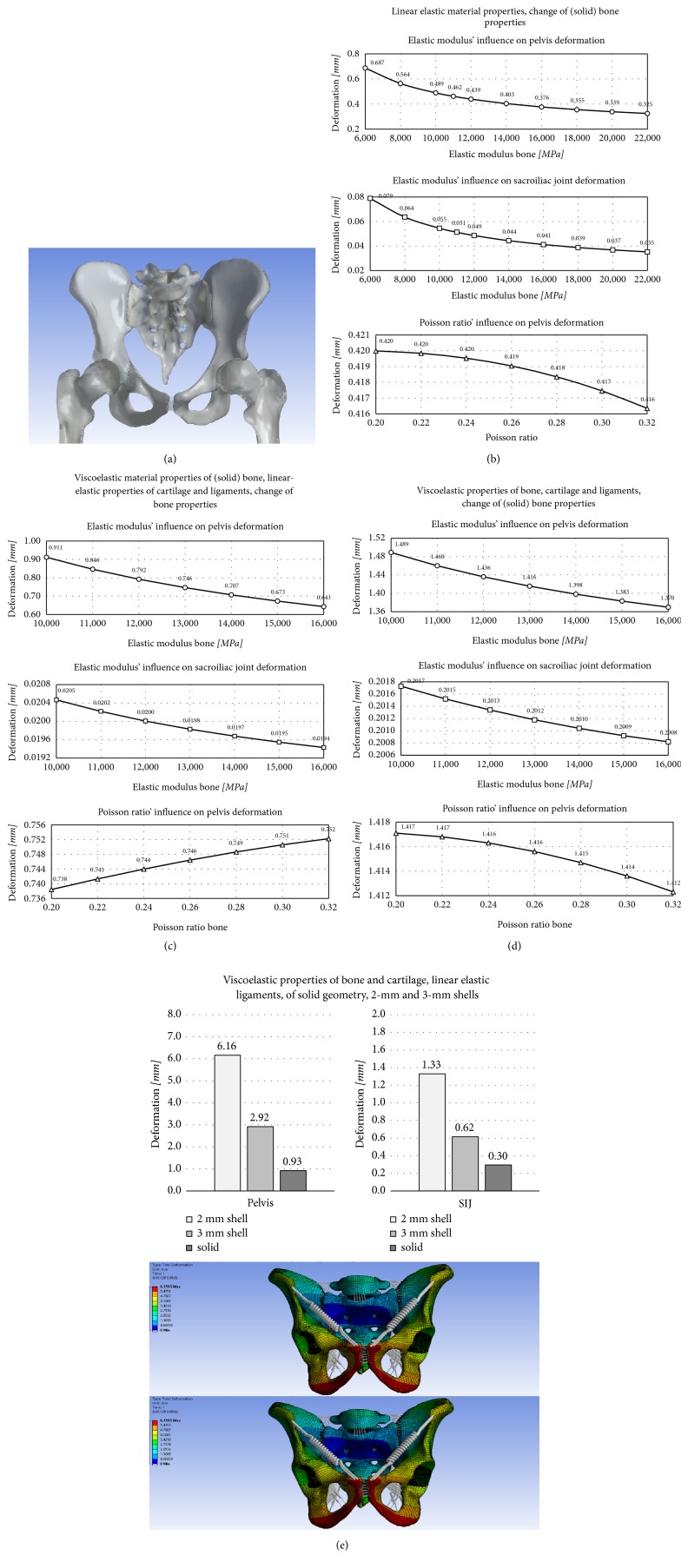
(a) Overview bone; parametric change of (b) linear elastic bone properties (solid), (c) viscoelastic bone (solid) with linear elastic cartilage and ligaments, (d) viscoelastic (solid) bone, cartilage and ligaments, and (e) change in bone geometry in a model (solid versus shell) with viscoelastic properties of bone and cartilage, linear elastic ligaments. The bottom figure shows the related deformation in a 2 mm shell model.

Bone material properties were changed parametrically between 6000 MPa and 22,000 MPa in 2000 MPa step sizes, with Poisson's ratio of 0.26. Poison's ratio was then changed between 0.20 and 0.32 with bone elastic modulus of 11,000 MPa. An increased elastic modulus decreased the deformation of the pelvis as well as the SIJ, with value ranges of 148.8-70.3% and 153.3–68.7%, respectively (Figures 2(a) and 2(b)). An inverted behavior was found for Poisson's ratio.

#### 3.1.2. Isotropic Viscoelastic Bone, Linear Elastic Cartilage, and Ligaments

For the simulations using viscoelastic material properties for (solid) bones and linear elastic material properties for the cartilage and ligaments, base line data were the same as above. Bone elastic modulus was varied between 10,000 and 16,000 MPa and Poisson's ratio 0.20 to 0.32. An increased elastic modulus was related to decreased pelvis (122.1-86.2%) and SIJ deformation (103.5-98.0%), and increasing Poisson's ratio was related to minutely increased pelvis deformation (98.9-100.8%; Figure 2(c)). Overall deformation was 1.78x larger compared to the linear elastic model.

#### 3.1.3. Isotropic Viscoelastic Bone, Cartilage, and Ligaments

Material properties were allocated as above for bone and ligaments; cartilage elastic modulus was 4.5 MPa and Poisson's ratio was 0.21. Bone elastic modulus was varied between 10,000 and 16,000 MPa and Poisson's ratio between 0.20 and 0.32. Increased elastic modulus or Poisson's ratio decreased the overall deformation of the pelvis in the range of 105.2–96.8% and 100.1–99.7%, respectively. A similar indirect trend was observed at the SIJ with 100.2–99.8% (Figure 2(d)). Overall deformation increased by 3.4x compared to the linear elastic model. A significant component of the observed motion was caused by the cartilage and ligaments.

#### 3.1.4. Isotropic Viscoelastic Properties for Bone and Cartilage and Linear Elastic Properties for Ligaments: Comparison of Solid Geometry to 2 and 3 mm Shell

Using isotropic viscoelastic material properties for all tissues, bone geometry was altered, creating 2 mm and 3 mm shell models of a constant thickness throughout all osseous structures of the pelvis. It could be found that pelvis and SIJ motion increased for the 2 mm and 3 mm model with a 662.4% and 443.3% change in the 2 mm model and a 314.0% and 200.7% change in the 3 mm model compared to the linear elastic model (Figure 2(e)). A significant component of the overall deformation came from the bones in the shell model, contributing to cartilage and ligament deformation.

#### 3.1.5. Summary: Bones

In summary, elastic modulus changes were more decisive than Poisson ratio changes, and deformations were larger using the viscoelastic compared to the linear elastic model, in particular with bone shell geometry. Deformations remained in the submillimeter range if bone and/or cartilage were modeled as linear elastic; SIJ motion was two magnitudes lower than pelvis motion. Viscoelastic properties gave results more similar to the cadaveric validation data.

### 3.2. Cartilage

#### 3.2.1. Isotropic Linear Elastic Model: Parametric Change of Cartilage Properties

**Figure 3:**
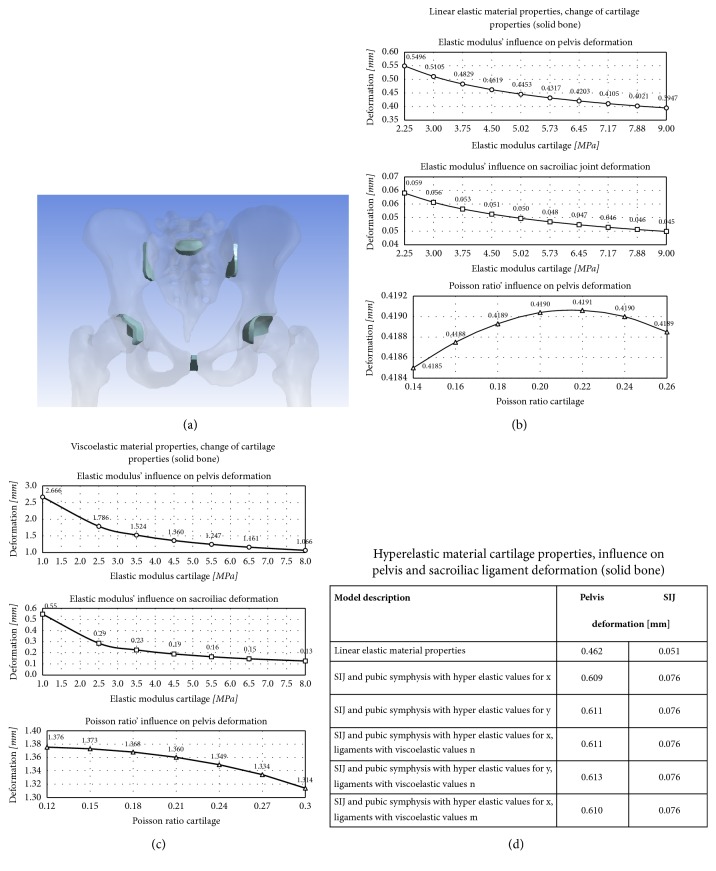
(a) Overview cartilage; parametric change of (b) linear elastic cartilage, (c) viscoelastic cartilage, and (d) hyperelastic cartilage (all solid bone).

Cartilage elastic modulus was changed in the range of 2.25 and 9.00 MPa in 1.125-MPa steps, from the base line of 4.50 MPa and Poisson's ratio of 0.20. Similarly, with cartilage elastic modulus kept constant at 4.50 MPa, Poisson's ration was changed between 0.14 and 0.26 in 0.02 increments. Elastic modulus increase was related to decreased pelvis and SIJ motion, with value ranges of 119.0-85.5% and 115.7-88.2%, respectively (Figures 3(a) and 3(b)). The influence of Poisson ratio on pelvis deformation was minute and nonlinear, with a maximum displacement at a ratio of 0.22. Deformations remained in the submillimeter range irrespective of the value changes.

#### 3.2.2. Isotropic Viscoelastic Bone and Cartilage

Bone elastic modulus and Poisson's ratio were set at 13,000 MPa and 0.26, respectively, and cartilage elastic modulus was varied between 1 and 8 MPa from the base line of 4.5 MPa, and Poisson's ratio was varied between 0.12 and 0.30. Decreased elastic modulus or Poisson's ratio increased overall deformation of the pelvis, with values ranging between 196.0–78.4% and 101.2–96.6%, respectively (Figure 3(c)). A similar inverted trend was observed at the SIJ (289.5–68.4%). Deformations increased by 3.3x compared to the linear elastic material model. A significant component of the observed motion was related to the cartilage and ligaments.

#### 3.2.3. Isotropic Hyperelastic Material Properties Cartilage Properties

Hyperelastic modeling was conducted for the sacroiliac joint and pubic symphysis, using different combinations shown in Table 3. The Mooney-Rivlin theory was applied, with software-based constants C1 of 4.1 x 10^6^ Pa and C2 of 4.1 x 10^5^ Pa based on experimental tensile measurements. Overall pelvis deformation comparing hyperelastic to linear elastic models was 0.61 mm versus 0.46 mm (32.2 % increase) and SIJ deformation 0.076 mm versus 0.051 mm (48.2 % increase), respectively.

#### 3.2.4. Summary: Cartilage

In line with the results of the bone, elastic modulus changes were more decisive than Poisson ratio changes, and deformations were larger using the viscoelastic compared to the linear elastic model. Viscoelastic properties gave more realistic results, whereas hyperelastic modeling gave minute changes in minimal deformations.

### 3.3. Ligaments

#### 3.3.1. Isotropic Linear Elastic Material Properties: Parametric Change of Ligament Properties

**Figure 4:**
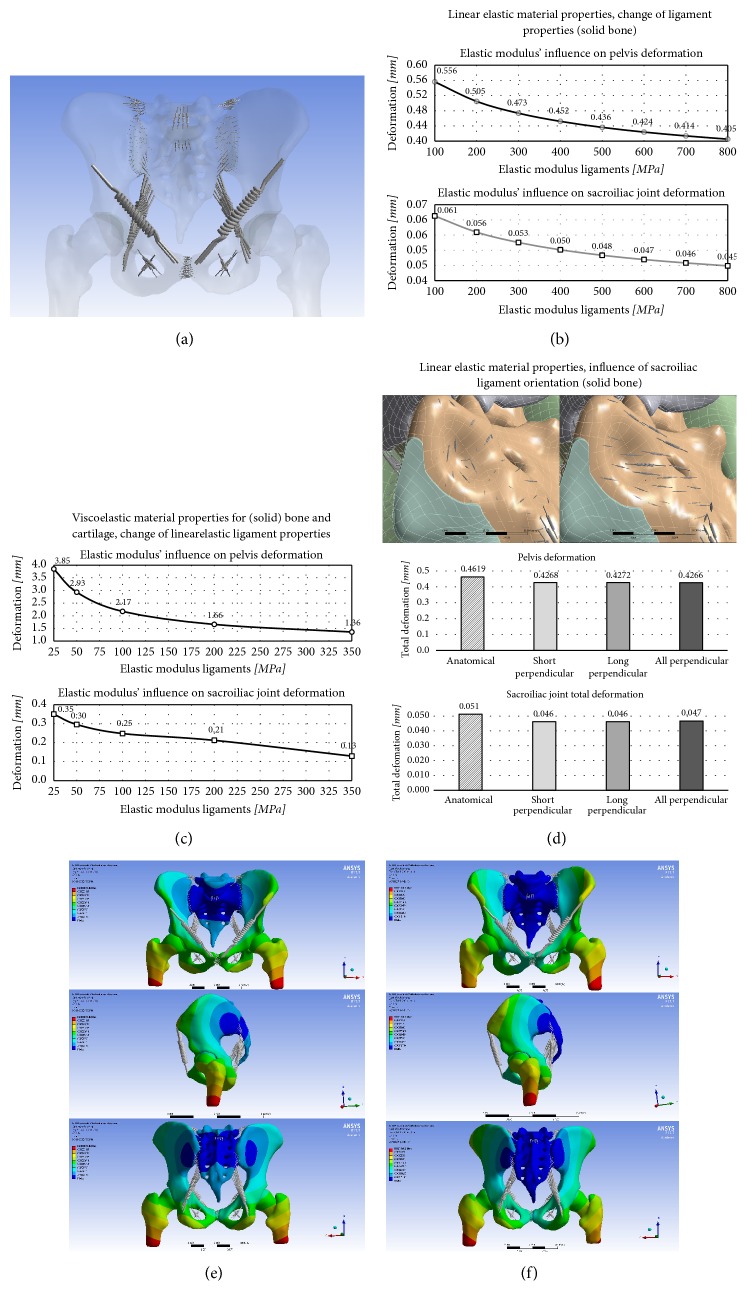
(a) Overview ligaments, (b) parametric analysis in linear elastic model, (c) deformations at a linear elastic model (top: anterior view, center: lateral view, and bottom: posterior view), (d) parametric analysis in viscoelastic model, (e) deformations at a viscoelastic model (top: anterior view, center: lateral view, and bottom: posterior view), (f) influence of sacroiliac ligament orientation. Global and local deformations were larger in the viscoelastic model compared to the linear elastic model, and in line with the real case scenario loads were distributed more homogeneously.

Ligament elastic modulus was varied between 100 and 800 MPa, with bone and cartilage properties left unchanged. Overall pelvis deformation and SIJ deformation changed in the range of 120.2–87.6% and 119.4-87.4% (Figures 4(a)–4(c)), respectively, and the overall deformation was minute.

#### 3.3.2. Viscoelastic Material Properties for Bone and Cartilage, Parametric Change of Ligament Properties

Elastic modulus and Poisson's ratio were set to be 13,000 and 0.26 for bone and 4.5 and 0.21 for cartilage, respectively. Prony volumetric relaxation data was used according to literature [38, 39]. Increasing elastic moduli were related to decreased deformation at the pelvis and SIJ, with changes between 201.0–71.0 and 152.2–56.5%, respectively (Figures 4(d) and 4(e)). The effects of parametric change were more pronounced in the viscoelastic compared to the linear elastic model.

#### 3.3.3. Isotropic Linear Elastic Material Properties: Altered SIJ Ligament Fiber Orientation

With solid bones, cartilage, and ligaments being allocated isotropic and linear elastic material properties, the alignment and implementation of the interosseous and posterior SIJ ligaments (here separated as short and long ligaments according to their length) were studied regarding their influence on pelvis and SIJ deformation. Overall changes induced by altered ligament orientation caused less than 10% change in overall motion, and this motion remained minute with less than 0.5 mm for the pelvis and less than 0.06 mm for the SIJ (Figure 4(f)).

### 3.4. Validation Results for the Viscoelastic Model

Comparison of the multiaxial movements from the cadaveric experiments (500-N load application) with the in silico results yielded the following results: Pelvis deformation was 0.93, 2.92 and 6.16 mm for the solid, and 3 mm and 2 mm FE models at 500 N, respectively. Pelvis deformation observed in the cadaveric experiments averaged 2.92 mm. SIJ deformation was 0.30, 0.62 and 1.33 mm for the solid and 3 mm and 2 mm FE models at 500 N, respectively. SIJ deformation observed in the cadaveric experiments averaged 0.31 mm. Regression analysis: R^2^ was 0.69, 0.99, and 0.56, for the 2 mm shell, 3 mm shell, and the solid bone model, respectively. More detail on the load deformations in the cadaveric pelvises can be found in Klima et al. and Hammer et al. [40, 41].

## 4. Discussion

This study investigated the effects of a variety of altering material properties, material laws, and different geometries on the load deformation of the human osteoligamentous pelvis, with the primary objective to adjust this model to recently obtained load deformation data in a complementary physiological loading setting in an inverted validation approach. It could be shown that alterations in geometry, material models, and mechanical data have significant influence on the overall deformation of the pelvis as well as the SIJ.

FE simulations have become an important method to research musculoskeletal tissues, involving healthy and pathologically altered biomechanics. At the pelvis, FE models are meanwhile used beyond basic science research questions to understand the mechanisms of pelvic ring injury and potential failure sites of tissues [1, 14, 42], clinical diagnostics around the painful SIJ [7, 43], and both nonsurgical [11] and surgical [4, 12, 13, 15, 16, 44] treatment. Accurate modeling is of expressive importance, as even implant-related research has increasingly started to use the FE method [12, 45].

Lindsay and coworkers [15, 16], based on a model of Ivanov et al. [44], have assessed in detail the mechanics of the SIJ and the adjacent lumbar spine following the surgical fusion of the posterior pelvis by means of triangular implants, underlining the need of FE models to answer parametrically clinical research questions. Such FE models can be run numerous times, assessing the mechanics of different surgical interventions in an anatomically highly standardized model. Such experiments would be impossible using cadaveric tissues due to large variations in the anatomy and bone density in humans. Recent research based on this model has furthermore helped to understand the effects of different leg length onto SIJ biomechanics [43] and provided evidence towards gender-specific loading of the SIJ cartilage and in particular of the surrounding ligaments [42].

The outcomes of FE modeling are crucially dependent on precise morphological and mechanical data and accurate loading conditions. An important challenge in particular at the pelvis is the fact that three joints are merged horizontally (pubic symphysis and bilateral SIJ) and three joints vertically (lumbosacral transition, SIJ, and hip joints), making this chain of joints challenging to understand for the biomechanicist and the clinician equally. Majority of the existing FE models do not represent physiological load deformation of the pelvis, in which positional changes of the pelvic ring itself are part of the overall movement.

The pelvis is moreover difficult to model due to the heterogeneous, anisotropic, and viscoelastic nature of the tissues, which is why most groups limit their FE pelvises to osteoligamentous structures [1, 2, 4–6, 9–11, 14, 46] or even just bones [3, 13]. Moreover, accurate load deformation data is missing in particular for the ligaments of the pelvis. The FE model presented here does not address each of these shortcomings, but does make attempt to demonstrate which factors might be influential to come close to validation data of physiological pelvis and SIJ motion in the two-leg standing scenario.

This given FE study has underlined the importance of experimental validation of computational results and in reverse shown the opportunity to trial which extend the various material properties resembling the actual* in vitro* situation.

For bone, elastic modulus changes were more decisive than Poisson ratio changes, and deformations were larger using the viscoelastic compared to the linear elastic model, in particular with bone shell geometry. Pelvis and SIJ deformations remained in the submillimeter range if bone and/or cartilage were modeled as linear elastic, and SIJ motion was usually two magnitudes lower than pelvis motion. Consequently, hypothesis A can be accepted. Viscoelastic properties gave more similar results as in the cadaveric experiments than linear elastic ones, approving hypothesis B. These data are in line with the results of Dalstra et al. [47] and Anderson et al. [3] who proposed region-dependent material data for the pelvic bones. Most existent studies but, however, use isotropic homogeneous material models in favor of efficient computing times [1, 2, 4, 7, 9, 12–14, 48–50]. A commonly introduced detail in the modeling is the separation of cortical and cancellous bone [4, 5, 7, 9, 12, 13, 48–52], though experimental evidence is to date lacking on the benefit this inclusion gives to the accuracy of the modeling.

For cartilage, increased elastic modulus or Poisson's ratio of cartilage decreased pelvis and SIJ deformation, with the elastic modulus being more decisive. The overall change of pelvis and SIJ motion was minute and at least one magnitude smaller at the SIJ compared to the pelvis. The cartilage of the SIJ is not consistently modeled in existing research and there is controversy regarding the importance of modeling the SIJ as a synovial joint. Hao et al. [6] in their study excluded the cartilage and Watson et al. [10] in their parametric analysis found no significant influence of modeling the SIJ cartilage as a mobile structure. In contrast to this, in another recent study, Shi et al. [9] found that including the SIJ as a synovial and mobile structure may increase the overall SIJ displacement by almost 100%.

Effects of parametric change of ligament properties were more pronounced in the viscoelastic compared to the linear elastic model. Maximum variations between ~50% and 200% were observed for the deformations. Surprisingly little influence was found for the interosseous and posterior SIJ ligament orientation in overall movement. Existing research on the topic pays differing attention to modeling of the ligaments of the pelvis. Though ligament orientation of these ligaments did not identify as a major driver of altered load deformation, a number of parametric analyses underline the importance of accurate ligament modeling [2, 5, 6], regarding both material properties and material models.

*Limitations*. A couple of limitations need to be addressed for the given study. First, the results presented here were derived from an individual model representing a male osteoligamentous pelvis. Though the osseous geometry was carefully selected to be equally representative for female pelvises, this is yet to be substantiated. Models including larger anatomical areas, e.g., as the model of Ivanov et al., would be beneficial [42, 44]. Furthermore, this study did not assess the impact of anisotropy or heterogenic material properties, though published research on the pelvic bones are indicative of this being an important factor to be modeled. Also, the coordinate system used here was different from the coordinate system recommendations of the ISB. Though this forms a shortcoming of the given study, the choice of coordinate system was done in line with previous research, our own work [40, 41], and the coordinate system used clinically by SIJ surgeons, orthopedics, and pain therapists.

*Summary. *This parametric study has assessed the influence of altered mechanical properties, material models, and geometries of the osteoligamentous pelvis in an anatomically detailed and experimentally validated model. It could be shown that geometry and material models are more decisive for bone and mechanical properties for both linear elastic and viscoelastic cartilage and ligament models. Fiber orientation of the interosseous and posterior SIJ ligaments had surprisingly little overall influence. The alterations in mechanical properties shown here may serve as rough estimates for age-dependent changes in bone, cartilage and soft tissue mechanics, interindividual variation, and varying geometries in order to serve as a basis for future simulations.

## Figures and Tables

**Table 1:** Number of nodes, elements, and element type for the different geometries.

**Model type**	**Number of nodes**	**Number of elements**	**Type of elements**

Solid model	151,642	87,233	tetrahedral

2-mm shell	282,581	195,611	quadrilateral, trilateral (dominant)
3-mm shell			

**Table 2:** Assigned material properties for linear elastic and viscoelastic models, obtained from literature [29–36].

**Material properties**	**Linear elastic**	**Viscoelastic**
Bone		
Elastic modulus	11 x 10^3^ MPa	13 x 10^3^ MPa
Variation elastic modulus	6 - 22 x 10^3^ MPa	10 - 16 x 10^3^ MPa
Poisson ratio	0.26	0.26
Variation poisson ratio	0.20 - 0.32	0.20 - 0.32
Bulk modulus		9.03 x 10^3^ MPa
Shear modulus		5.16 x 10^3^ MPa
Prony volumetric relaxation		
Relative modulus		0.713
Relaxation time		6.9 sec
Density		1640 kg/m^3^

Cartilage		
Elastic modulus	4.5 MPa	4.5 MPa
Variation elastic modulus		1 - 8
Poisson ratio	0.20	0.21
Bulk modulus		2.59 x 10^3^ Pa
Shear modulus		1.86 x 10^3^ Pa
Prony volumetric relaxation		
Relative modulus		0.713
Relaxation time		6.9 sec
Variation	0.14 - 0.26	0.12 - 0.30

Ligament		
Elastic modulus	350 MPa	350 MPa
Variation	100 – 800 MPa	100 - 800 MPa

**Table 3:** Changes in material laws for ligaments, sacroiliac joint (SIJ) and pubic symphysis and resulting deformation (x and y refer to the ISB standard axes).

**Model description**	**Pelvis**	**SIJ**
	**deformation [mm]**	
Linear elastic material properties	0.462	0.051
SIJ and pubic symphysis with hyper elastic values for x	0.609	0.076
SIJ and pubic symphysis with hyper elastic values for y	0.611	0.076
SIJ and pubic symphysis with hyper elastic values for x, ligaments with viscoelastic values n	0.611	0.076
SIJ and pubic symphysis with hyper elastic values for y, ligaments with viscoelastic values n	0.613	0.076
SIJ and pubic symphysis with hyper elastic values for x, ligaments with viscoelastic values m	0.610	0.076

## Data Availability

The data used to support the findings of this study are available from the corresponding author upon request.
